# Circular RNA Rftn1 Promotes Cardiac Hypertrophy In Vitro and In Vivo by Sponging miRNA‐1192 to Upregulate Tripartite Motif Protein 25 and 41

**DOI:** 10.1111/jcmm.70892

**Published:** 2025-10-13

**Authors:** Guangcheng Liu, Haipeng Zhang, Jingdai Zhang, Hao Qian, Liang Wang, Lianfeng Chen, Zhujun Shen

**Affiliations:** ^1^ Department of Cardiology Medicine Peking Union Medical College Hospital, Chinese Academy of Medical Science and Peking Union Medical College Beijing China

**Keywords:** cardiac hypertrophy, competing endogenous RNA, miR‐1192, *Mus musculus* circular RNA Rftn1, tripartite motif

## Abstract

To verify the role of circular RNA circRftn1 in cardiac hypertrophy and elucidate its potential mechanism via the microRNA‐1192‐tripartite motif 25 and 41 (miR1192‐TRIM25/TRIM41) axis. A mouse model of cardiac hypertrophy was established via abdominal aortic coarctation surgery. An in vitro cell model was generated using neonatal mouse ventricular cardiomyocytes (NMVCs) co‐cultured with angiotensin II. Differentially expressed circular RNAs (circRNAs) were identified using next‐generation sequencing, and potential target microRNAs (miRNAs) and downstream messenger RNAs (mRNAs) were predicted using bioinformatic analysis. Reverse transcription quantitative polymerase chain reaction and western blotting were used to evaluate the expression of myocardial‐associated molecules at the transcriptional and translational levels, respectively. Expression of regulatory molecules was assessed after transfection with small interfering RNAs (siRNAs) or co‐culture with miRNA mimics. Dual‐luciferase reporter assays were performed to validate interactions between circRNAs and miRNAs. circRftn1 expression was significantly elevated both in vivo and in vitro. Concurrently, the expression of miR‐1192 was decreased, whereas its target mRNAs TRIM25 and TRIM41 were markedly upregulated. Knockdown of circRftn1 via siRNA transfection reversed cardiac hypertrophy and led to opposing expression trends of miR‐1192 and TRIM25/TRIM41. Dual‐luciferase reporter assays confirmed the sponge‐like interaction between circRftn1 and miR‐1192. By co‐culturing NMVCs with miR‐1192 mimics, its targets TRIM25/TRIM41 showed significant decreases. Moreover, NF‐κB signalling pathway appeared to be involved, as the expression of the p65 subunit paralleled that of circRftn1. circRftn1 may promote cardiac hypertrophy by modulating the miR‐1192‐TRIM25/TRIM41 axis and NF‐κB p65 pathway potentially serving as a downstream pathway.

**Abbreviations**
Ang II: angiotensin II
CeRNA: competing endogenous RNA
circRNA: circular RNA
HF: heart failure
miRNA: micro RNA
ncRNA: non‐coding RNA
NMVCs: neonatal mice ventricle cardiomyocytes
TRIM: tripartite motif

## 1 Introduction

Pathological cardiac hypertrophy is a maladaptive form of cardiac remodelling that occurs in variable clinical conditions, including hypertension and cardiomyopathy, and progressively advances to heart failure (HF) [1, 2, 3], which remains a major global public health issue with high morbidity and mortality areas [4, 5]. A critical unmet need lies in the sensitive detection of early‐stage HF and effective reversal of myocardial enlargement and fibrosis [6, 7]. Multiple stimuli and signalling mechanisms have been implicated in pathological cardiac hypertrophy [8, 9, 10] beyond pressure overload only, including alterations at the genomic and transcriptional levels such as impaired calcium (Ca^2+^) handling [11], mitochondrial dysfunction [12], oxidative stress [13, 14], and N^6^‐methyladenosine methyletation [15, 16]. Researchers continue to explore these complex mechanisms, and next‐generation sequencing technologies have enabled breakthroughs, notably by highlighting the roles of non‐coding RNAs (ncRNAs) in cardiovascular regulation.

With advances in transcriptomic analysis, ncRNAs—including the recently recognised class of circular RNAs (circRNA)—are gaining increasing attenion [17, 18, 19]. Endogenous circRNAs have been implicated in various aspects of cardiovascular diseases [4, 20, 21, 22, 23], spanning processes from cardiac embryogenesis to both physiological and pathological regulation [24, 25, 26]. As important regulators, circRNAs can function as microRNA (miRNA) sponges, modulate messenger RNA (mRNA) translation, or directly participate in gene transcription as transcription factors [27, 28]. Typically generated through non‐canonical back‐splicing of parental genes, circRNAs act as competing endogenous RNAs (ceRNAs) by sequestering specific miRNAs and thereby influencing downstream gene expression [29, 30]. For example, circ_000203 promotes expression of fibrosis‐related genes in cardiac fibroblasts by suppressing miR‐26b‐5p, contributing to cardiac fibrosis [31], whereas heart‐related circRNA protects the heart from pathological hypertrophy by targeting miR‐223 [32]. To date, several ceRNA networks have been identified as either stimulatory or inhibitory in cardiovascular pathologies such as atherosclerosis (such as circ_0026218/miR‐338‐3p [33], and circCHFR/miRNA‐15b‐5p [34]) and heart failure (such as circNfix/miR‐145‐5p [35], CDR1as/miRNA‐671‐5p [36], circSlc8a1/miR‐133a [37], and circmiRs/miR‐132 [38]). These findings highlight the therapeutic potential of circRNA/miRNA axes in treating cardiac hypertrophy, drawing substantial research interest.

In this study, we identified a novel circular RNA, *Mus musculus* circ_0004641 (circRftn1), in mouse myocardium and proposed a new ceRNA network: the circRftn1/miRNA‐1192 axis. Both loss‐ and gain‐of‐function experiments demonstrated the regulatory role of the circRftn1/miR‐1192 axis in modulating myocardial hypertrophy in vivo and in vitro, which is through the regulation of downstream target mRNA—tripartite motif‐containing protein 25 (TRIM25) and tripartite motif‐containing protein 41 (TRIM41). In addition, we explored the involvement of the nuclear factor kappa‐light‐chain‐enhancer of activated B cells (NF‐κB) signalling pathway as a potential downstream effector of this ceRNA network.

## 2 Materials and Methods

### 2.1 Establishment and Evaluation of an Animal Model

Healthy specific pathogen‐free C57BL/6 male mice (6–8 weeks old, 20–22 g) were obtained from Experimental Animal Technology Co. Ltd., Weitonglihua (Beijing, China). All animal care and experimental procedures were approved by the Animal Research Ethics Committee of Peking Union Medical College Hospital (PUMCH, Beijing, China; approval number: XHDW‐24‐86). Abdominal aortic coarctation (AAC) surgery, a widely used method for inducing cardiac hypertrophy [39], was performed as described in detail in Supporting Information S1.

Cardiac function was evaluated 4 weeks after the AAC procedure using transthoracic echocardiography with the Vevo 2100 Imaging system (VisualSonics, Canada). Mice were anaesthetised with isoflurane (2.5% for induction, 1.0% for maintenance), and heart rates were maintained between 400 and 450 beats/min. Left ventricular parameters during diastole and systole were assessed using M‐mode and Doppler‐mode imaging, as detailed in Supporting Information S1.

### 2.2 Histochemistry Analysis

Following euthanasia, mouse hearts were excised, fixed in 4% paraformaldehyde, dehydrated, and paraffin‐embedded using standard procedures. The paraffin blocks were sectioned at 4–5 μm thickness. Cardiac cross‐sections were stained with haematoxylin–eosin (H&E, Servicebio, China) and Sirius Red (Sigma, USA) to evaluate myocardial morphology and fibrosis. Fluorescein isothiocyanate‐conjugated wheat germ agglutinin (WGA) (Sigma, L4895, 1:500, USA) was used to outline cardiomyocyte membranes, and 4′,6‐diamidino‐2‐phenylindole (DAPI) (Servicebio, 1 μg/mL) was used to label cell nuclei. Images were acquired using a Leica microscope (Leica, Wetzlar, Germany) and analysed using CaseViewer 2.4 software.

### 2.3 RNA Fluorescence In Situ Hybridisation (FISH)

FISH was performed to visualise the intracellular localisation of circRftn1 in cardiomyocytes using the FISH Tag RNA Multicolor Kit (Invitrogen, USA). Briefly, myocardial tissue was fixed in 4% paraformaldehyde at room temperature for 30 min and permeabilised with 0.5% Triton X‐100 (Biosharp, Hefei, China) for 1 h. Samples were pre‐incubated with hybridisation buffer (component C:A = 1:99) at 37°C for 30 min, followed by overnight hybridisation with circRftn1‐specific probes (RiboBio, Guangzhou, China) at 37°C. The following day, the sections were sequentially washed with saline‐sodium citrate buffer at 42°C. Nuclei were counterstained with DAPI (Beyotime, Beijing, China) for 15 min. Imaging was performed using a scanning laser confocal microscope (Leica, Wetzlar, Germany).

### 2.4 Cell Culture and Transfection

Neonatal mouse ventricle cardiomyocytes (NMVCs) were isolated from neonatal C57BL/6 mice (aged 1–3 days). Ventricle tissues were cut into 1 mm^3^ pieces and digested using 0.1% collagenase II (Gibico, China). NMVCs were cultured in Dulbecco's Modified Eagle Medium/Nutrient Mixture F‐12 (DMEM/F12) (Thermo Fisher Scientific, USA) supplemented with 100 U/mL penicillin, 100 mg/mL streptomycin, and 10% fetal bovine serum (Hyclone, USA). Cells were maintained at 37°C in a humidified atmosphere with 5% CO_2_ and 95% O_2_. To induce a hypertrophic phenotype, NMVCs were treated with 10^−7^ M angiotensin II (Ang‐II, Selleck, USA) for 48 h.

Small interfering RNA (siRNA) targeting mmu_circRftn1 (5 nmol/L, RiboBio, China) was transfected into NMVCs, followed by co‐incubation with Ang II or DMSO for 48 h. The sequences of siRNA of circRftn1 are as follows: CATGGCCAATGGTGCAGGA. Overexpression mimics targeting mmu_miR‐1192 (50 pmol/L, Sangon Biotech, China) were co‐cultured with NMVCs using Ang II or dimethyl sulfoxide (DMSO) for 48 h. The sequence of the miR‐1192 mimics was: AAACAAACAAACAGACCAAAUU.

### 2.5 RNA Extraction, Library Preparation and circRNA Sequencing

Total RNA was extracted from mouse left ventricular tissues using TRIzol reagent (Invitrogen, USA). RNA quality and integrity were assessed using the RNA Nano 6000 Assay Kit and the Agilent Bioanalyzer 2100 system (Agilent Technologies, CA, USA). Ribosomal RNA (rRNA) was removed using the Ribo‐Zero Gold Kit (Epicenter Technologies, USA). Sequencing libraries were prepared using the NEBNext Ultra RNA Library Prep Kit for Illumina (New England Biolabs, USA) according to the manufacturer's protocol. Sequencing was performed on the Illumina HiSeq 4000 platform.

### 2.6 Identification of circRNA Sequences and Bioinformatic Analysis

Raw sequencing data were filtered to ensure quality and reliability. Clean reads were aligned to the reference genome using **HISAT2** software [40] to obtain accurate localisation information. Candidate circRNAs were identified using the **Find_Circle** algorithm [30] and the **circRNA Identifier** algorithm [41] (detailed in Supporting Information). The trimmed mean of M‐values (**TMM) algorithm** was applied to normalise read count data. For samples with biological replicates, differential expression was analysed using the **DESeq2** algorithm [42]. For samples without replicates, the **edgeR** algorithm [43] was used following TMM normalisation. Differentially expressed circRNAs were screened using thresholds of adjusted *p* value (*p*_adj < 0.05) and log_2_fold change (log_2_FC > 1.0). Visualisation of results was performed using volcano plots and heatmaps. Gene Ontology (GO) and Kyoto Encyclopedia of Genes and Genomes (KEGG) enrichment analyses were performed using the **clusterProfiler** package [44]. Potential miRNA binding sites were predicted using **miRanda** software [29] and the **miRDB** database (http://mirdb.org/miRDB). Predicted miRNA–mRNA and circRNA–miRNA interactions were visualised using Cytoscape software (version 3.10; http://www.cytoscape.org/).

### 2.7 Western Blot Analysis

Total protein was extracted from heart tissues or cardiomyocytes. Protein concentration was determined using a bicinchoninic acid Protein Assay Kit (Beyotime, Shanghai, China). A total of 40 μg of protein was subjected to electrophoresis using a 10% sodium dodecyl sulfate‐polyacrylamide gel and transferred onto a polyvinylidene fluoride membrane. Membranes were blocked with 5% skimmed milk at room temperature for 2 h and incubated overnight at 4°C with the following primary antibodies: β‐myosin heavy chain (β‐MHC, 1:1000; ab172967, Abcam, UK), atrial natriuretic peptide (ANP, 1:2000; 27426‐1‐AP, Proteintech, USA), tripartite motif‐containing 25 (TRIM25, 1:1000; 12573‐1‐AP, Proteintech, USA), tripartite motif‐containing 41 (TRIM41, 1:1000; 18468‐1‐AP, Proteintech, USA), phosphorylated nuclear factor kappa B (p‐NFκB, 1:1000; 3033, Cell Signaling Technology [CST], USA), nuclear factor kappa B (NFκB, 1:1000; 8242, CST, USA), transforming growth factor‐beta 1 (TGF‐β1, 1:1000; ab179695, Abcam, UK), (SMAD1/5, 1:1000; ab300164, Abcam, UK), phosphorylated SMAD 1/5 (p‐SMAD1/5, 1:1000; ab9516, CST, USA), SMAD2/3 (1:1000; 3102, CST, USA), phosphorylated SMAD2/3 (p‐SMAD2/3, 1:1000; 8828, CST, USA), fibronectin 1 (FN1, 1:1000; ab2413, Abcam, UK), glyceraldehyde 3‐phosphate dehydrogenase (GAPDH, 1:10,000; ab181602, Abcam, UK). Following primary antibody incubation, membranes were incubated with species‐appropriate secondary antibodies (rabbit: 1:5000; 5220‐0336, KPL, USA or mouse: 1:10,000; 5220‐0341, KPL, USA) at room temperature for 1 h. GAPDH was used as a loading control. Protein bands were visualised using the GelView 6000Pro system (BLT, China), and band intensity was quantified using Image J software.

### 2.8 Quantitative Real‐Time PCR (qRT–PCR)

Total RNA was extracted from left ventricular tissues or NMVCs with TRIzol reagent (Invitrogen, USA). Reverse transcription was performed using a reverse transcription kit (TOYOBO, FSQ‐101, Japan). qRT–PCR was conducted using the ChamQ Universal SYBR qPCR Master Mix Kit (Q711‐03; Vazyme, China) on a CFX96 detection system (Bio‐Rad, USA). Gene expression was calculated using the 2^−ΔΔCt^ method. GAPDH was used as the internal control for normalisation. Primer sequences are listed in the Supporting Information.

### 2.9 Dual‐Luciferase Reporter Assay

To evaluate the direct interaction between circRftn1 and miR‐1192, the 3′ untranslated region (3′UTR) of circRftn1, containing either the conserved miR‐1192 binding sites (wild‐type) or mutated sequences, was synthesised and subcloned into the psiCheck2 dual‐luciferase reporter vector (Promega, Beijing, China). Human embryonic kidney‐293T cells (3 × 10^5^ cells per well in 12‐well plate) were co‐transfected with 200 ng of recombinant reporter plasmid, 20 ng of pRL‐TK vector (internal control; Promega, Madison, WI, USA), 200 ng of either pDsRed2‐N1 or pDsRed2‐miR‐192, and 50 nM of either circRftn1 mimic or mutant mimic. After 48 h, the culture medium was removed, and cells were washed twice with phosphate‐buffered saline. Passive Lysis Buffer (PLB, 100 mL per well) was added, and cells were gently shaken at room temperature for 15 min. Firefly luciferase (FL) and Renilla luciferase (RL) activities were measured using the Dual‐Luciferase Reporter Assay System (Promega, Beijing, China). The FL/RL ratio was used to assess circRftn1‐mediated regulation of gene expression. RL reporter vectors were used as an internal control.

### 2.10 Statistical Analysis

Data are presented as the mean ± standard error of the mean. For comparisons between two groups, an unpaired two‐tailed Student's *t*‐test was used. For comparisons among multiple groups, one‐way analysis of variance followed by Bonferroni post hoc tests was performed. All statistical analyses were conducted using GraphPad Prism version 9 (GraphPad Software, USA). A *p* value < 0.05 was considered statistically significant.

## 3 Results

### 3.1 Identification of Differentially Expressed circRNAs in AAC Models

**FIGURE 1:**
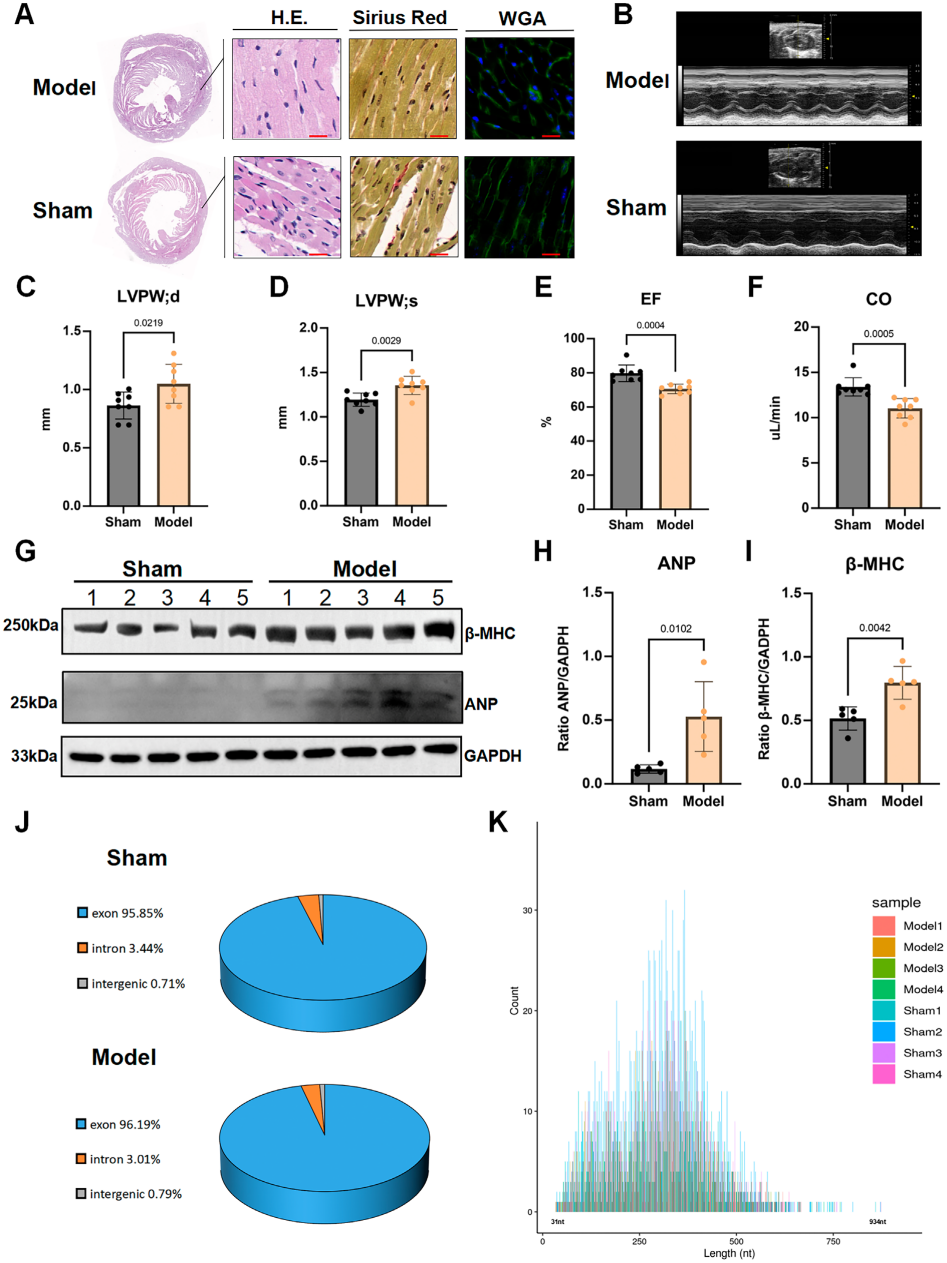
Construction of a cardiac hypertrophy mouse model of AAC. (A) Representative histopathology evaluation of cardiac hypertrophy: HE staining, Sirius Red staining, and WGA staining; (B) Representative M‐mode image of model and sham mice; (C–F) Echocardiography evaluation on cardiac parameters: Left ventricular posterior wall thickness, end‐diastolic/systolic (LVPW;d/LVPW;s); Left ventricular ejection fraction (LVEF), ejection fraction = (stroke volume/diastolic volume) × 100%; Cardiac output (CO) = stroke volume × heart rate, stroke volume = diastolic volume—systolic volume (*N* = 8); (G–I) Western blotting showed beta‐myosin heavy chain (β‐MHC) and atrial natriuretic peptide (ANP) overexpression (*N* = 5); (J) Class distribution of circRNAs in sham and model groups by next‐generation sequencing; (K) Length distribution of circRNAs. Scale bar is 100 μm. Data in (C–F), (H) and (I) are presented as mean ± SEM. For statistical analysis, a 2‐tailed unpaired *t*‐test was used for (C–F), (H) and (I). *p* < 0.05 was considered significant. β‐MHC, β‐myosin heavy chain; ANP, atrial natriuretic peptide; circRNA, circular RNA; CO, cardiac output; H&E, haematoxylin and eosin; LVEF, left ventricular ejection fraction; LVPW;d, left ventricular posterior wall thickness at end‐diastole; LVPW;s, left ventricular posterior wall thickness at end‐systole; NGS, next‐generation sequencing; SEM, standard error of the mean; WGA, wheat germ agglutinin.

We first established a C57B/L mouse model of myocardial hypertrophy through surgery at 8 weeks (8 mice per group: sham and model). To evaluate hypertrophy, H.E. staining, Sirius Red and WGA staining showed an increased hypertrophic cross‐sectional area in the myocardium of the model group (Figure 1A), along with an increased heart mass and enlarged ventricles (Figure S1A–D). Transthoracic echocardiography was performed to evaluate heart function concurrently. Compared to the sham group, the left ventricular posterior wall, interventricular septum and left ventricular volume were significantly increased in both systolic and diastolic phases in the AAC mice (Figure 1B–D; Figure S1E–K), while ejection fraction and cardiac output were sharply decreased (Figure 1E,F). Furthermore, western blotting was performed to detect cardiac biomarkers, and the results showed that β‐MHC and ANP were increasingly expressed, indicating cardiac hypertrophy (Figure 1F,G).

**FIGURE 2:**
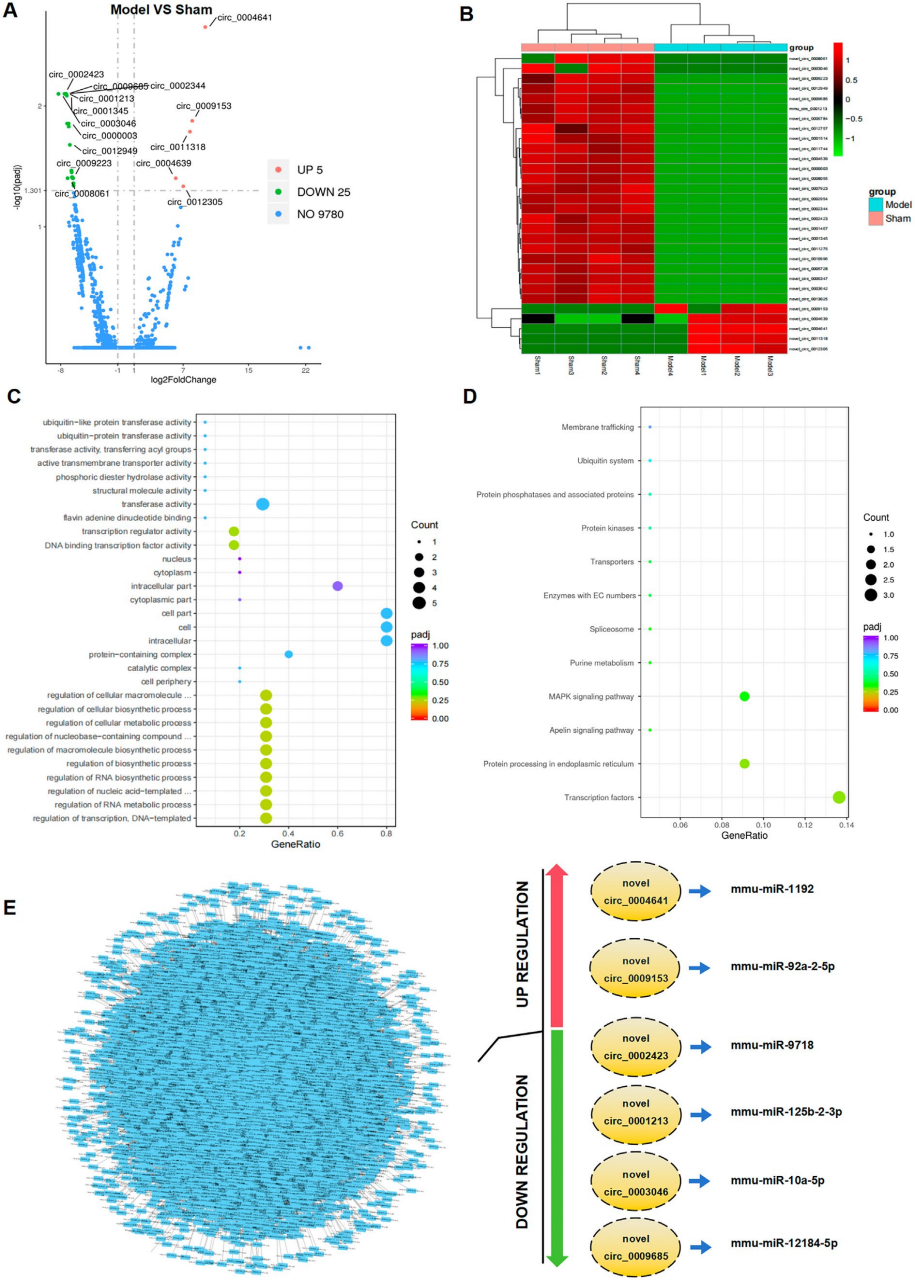
Distribution profiles of circRNAs. (A) The Volcano plot of DE circRNAs in sham and model mice. Shown are the top five upregulation and top 10 downregulated molecules; (B) The heatmap of DE circRNAs in sham and model mice; (C) GO analysis of the DE parental genes of circRNAs; (D) KEGG pathway enrichment analysis of the DE parental genes of circRNAs; (E) Network of circRNAs and miRNA; circRNAs in yellow and miRNA in black; green represents downregulation and red represents upregulation. circRNA, circular RNA; DE, differentially expressed; GO, gene ontology; KEGG, Kyoto Encyclopedia of Genes and Genomes; miRNA, microRNA.

After euthanizing the mice, a total of eight ventricular myocardium samples (four from the sham and model groups, respectively) were sent for circRNA sequencing (Figure 1H,I). We identified a total of 9813 circRNAs across the two groups (see Data Availability Statement). Based on their locations within host genes, the circRNAs were classified into three categories: 95.85% exonic, 3.44% intronic, and 0.71% intergenic in the sham group; and 96.19% exonic, 3.01% intronic, and 0.79% intergenic in the model group (Figure 1J; Figure S2A,B). The majority of the circRNAs ranged in length from 31 to 934 base pairs (Figure 1K) and were distributed across all chromosomes (Figure S2C). Among these, five circRNAs exhibited increased expression, while 25 circRNAs had decreased expression in the AAC mice with cardiac hypertrophy compared to the sham group (Figure 2A,B).

To gain further insight into the potential biological functions of these circRNAs, we annotated the parental genes of the circRNAs through functional enrichment analysis. GO biological process analysis revealed enrichment in transferase activity and ubiquitination (Figure 2C). KEGG pathway analysis showed that functional circRNAs were mainly enriched in transcription factors, mitogen‐activated protein kinase signalling pathway, and the ubiquitin system (Figure 2D). Next, targeted miRNAs were predicted using **miRanda** software, and an interaction diagram between circRNA and miRNA was constructed (Figure 2E). Based on the *p* value of expression, we specifically focused on six circRNAs for further study, including the top two upregulated and top four downregulated circRNAs (Table 1).

**TABLE 1:** Differential expressed gene of circular RNAs.

circRNA_ID	Log2 Fold Change	*p*_value	*p*_adj	Regulation	Chr	Source_gene_name	Gene_id	Start	End	Strand	Full_length	Spliced_length	Feature	Junction_read
novel_circ_0004641	9.708434	0.000000697	0.002224	Up	17	Rftn1	ENSMUSG00000039316	50354324	50362655	−	8331	595	exon: 50354325–50354528, exon: 50362265–50362655	240,144,433
novel_circ_0002954	−7.52196	0.00000764	0.007937	Down	14	—	n/a	10748553	10755291	−	6738	322	intergenic_region: 10748554–10748658, intergenic_region: 10755075–10755291	18,45,17,34
novel_circ_0009685	−7.41516	0.0000114	0.007937	Down	5	Thoc2l	ENSMUSG00000097392	104686691	104689128	+	2437	317	exon: 104686692–104686839, exon: 104687997–104688068, exon: 104689032–104689128	19,38,23,21
novel_circ_0002423	−7.47649	0.0000114	0.007937	Down	13	Ryr2	ENSMUSG00000021313	11839159	11844564	−	5405	442	exon: 11839160–11839295, exon: 11842420–11842603, exon: 11844443–11844564	38,46,9,20
mmu_circ_0001213	−7.38801	0.0000135	0.007937	Down	4	Ecpas	ENSMUSG00000050812	58861524	58885498	−	23974	1031	exon: 58861525–58861644, exon: 58864347–58864428, exon: 58877045–58877163, exon: 58879018–58879134, exon: 58885368–58885498	13,40,22,26
novel_circ_0003046	−8.26824	0.0000172	0.007937	Down	14	Kat6b	ENSMUSG00000021767	21566693	21567566	+	873	873	exon: 21566694–21567566	79,97,33
novel_circ_0002344	−7.31877	0.0000174	0.007937	Down	13	Pde4d	ENSMUSG00000021699	109876920	109909214	+	32294	353	exon: 109876921–109877112, exon: 109894111–109894147, exon: 109906068–109906141, exon: 109909165–109909214	15,31,16,33
novel_circ_0001345	−7.24862	0.0000206	0.008215	Down	11	Crk	ENSMUSG00000017776	75583022	75583558	+	536	444	exon: 75583023–75583151, exon: 75583244–75583558	25,36,14,18
novel_circ_0009153	8.11243	0.0000374	0.013271	Up	4	Fbxo42	ENSMUSG00000028920	140895023	140907843	+	12820	517	exon: 140895024–140895288, exon: 140898135–140898251, exon: 140907709–140907843	20,99,186
novel_circ_0000003	−7.03029	0.0000474	0.013957	Down	10	Tmtc2	ENSMUSG00000036019	105157256	105159498	−	2242	350	exon: 105157257–105157335, exon: 105158723–105158907, exon: 105159413–105159498	24,26,11,17
novel_circ_0012757	−7.20389	0.0000481	0.013957	Down	9	Atp2c1	ENSMUSG00000032570	105316539	105326087	−	9548	581	exon: 105316540–105316644, exon: 105319965–105320054, exon: 105320145–105320240, exon: 105322417–105322514, exon: 105323251–105323375, exon: 105326021–105326087	35,31,3,23
novel_circ_0003642	−7.0065	0.0000556	0.01479	Down	15	Oxct1	ENSMUSG00000022186	4076946	4087257	+	10311	393	exon: 4076947–4077082, exon: 4083197–4083346, exon: 4087151–4087257	16,44,7,20
novel_circ_0011318	7.832763	0.0000665	0.016318	Up	6	—	n/a	90575167	90613983	+	38816	345	intergenic_region: 90575168–90575367, intergenic_region: 90613839–90613983	107,29,79
novel_circ_0012949	−6.87058	0.000092	0.020968	Down	9	Zfp445	ENSMUSG00000047036	122685763	122686226	−	463	463	exon: 122685764–122686226	9,35,17,12
novel_circ_0004539	−6.67795	0.000161	0.034231	Down	17	Mapk14	ENSMUSG00000053436	28943717	28956064	+	12347	457	exon: 28943718–28943829, exon: 28944467–28944496, exon: 28944771–28944818, exon: 28947331–28947445, exon: 28947878–28947949, exon: 28955985–28956064	18,19,10,13
novel_circ_0008055	−6.65704	0.000175	0.034933	Down	2	Ttn	ENSMUSG00000051747	76680913	76714541	−	33628	573	exon: 76680914–76680988, exon: 76681899–76682108	18,19,8,15
novel_circ_0011744	−6.6528	0.000189	0.035412	Down	7	Ppfia1	ENSMUSG00000037519	144034821	144038945	−	4124	411	exon: 144034822–144034987, exon: 144035448–144035516	18,14,12,12
novel_circ_0010996	−6.61494	0.000227	0.03872	Down	6	Strip2	ENSMUSG00000039629	29939035	29953452	+	14417	400	exon: 29939036–29939162, exon: 29941838–29941938, exon: 29944425–29944491, exon: 29953348–29953452	9,43,7,11
novel_circ_0005728	−6.57749	0.000231	0.03872	Down	19	Sorbs1	ENSMUSG00000025006	40310236	40332883	−	22647	420	exon: 40310237–40310404, exon: 40328489–40328590, exon: 40329129–40329194, exon: 40332800–40332883	13,30,6,14
novel_circ_0001514	−6.61558	0.000243	0.038796	Down	11	Vezf1	ENSMUSG00000018377	87963882	87967200	+	3318	348	exon: 87965488–87965551, exon: 87967017–87967200	24,15,6,12
novel_circ_0006784	−6.49768	0.000298	0.039557	Down	1	Acadl	ENSMUSG00000026003	66876120	66880894	−	4774	329	exon: 66876121–66876207, exon: 66877438–66877565, exon: 66880781–66880894	8,23,12,12
novel_circ_0004639	6.093002	0.000298	0.039557	Up	17	Rftn1	ENSMUSG00000039316	50343968	50362655	−	18687	711	exon: 50343969–50344084, exon: 50354325–50354528, exon: 50362265–50362655	3,3,174,112,278
novel_circ_0008061	−7.14907	0.00031	0.039557	Down	2	Ttn	ENSMUSG00000051747	76684493	76712183	−	27690	646	exon: 76684494–76684562, exon: 76684731–76684811, exon: 76688248–76688331, exon: 76692694–76692763, exon: 76693144–76693203, exon: 76711902–76712183	35,28,20
novel_circ_0011275	−6.4849	0.000318	0.039557	Down	6	Exoc6b	ENSMUSG00000033769	84723882	84724766	−	884	255	intron: 84723883–84723956, intron: 84724586–84724766	16,24,8,8
novel_circ_0006347	−6.47065	0.000322	0.039557	Down	1	Atf6	ENSMUSG00000026663	170614914	170622339	−	7425	397	exon: 170614915–170615029, exon: 170616177–170616244, exon: 170621526–170621625, exon: 170622226–170622339	13,25,6,13
novel_circ_0009223	−6.53919	0.000322	0.039557	Down	4	Ube4b	ENSMUSG00000028960	149465639	149471673	−	6034	324	exon: 149469582–149469669, exon: 149471538–149471673	4,29,13,13
novel_circ_0001467	−6.42916	0.000377	0.043637	Down	11	Tns3	ENSMUSG00000020422	8468198	8482509	−	14311	551	exon: 8468199–8468303, exon: 8469123–8469188, exon: 8469441–8469570, exon: 8480872–8480947, exon: 8481704–8481764, exon: 8482397–8482509	15,24,6,10
novel_circ_0007923	−6.44279	0.000383	0.043637	Down	2	Stk39	ENSMUSG00000027030	68221257	68222596	−	1339	417	exon: 68221258–68221313, exon: 68221907–68222125, exon: 68222455–68222596	11,19,5,19
novel_circ_0013025	−6.41788	0.000415	0.045629	Down	9	Tbx20	ENSMUSG00000031965	24661607	24677070	−	15463	345	exon: 24661608–24661684, exon: 24670022–24670180, exon: 24676962–24677070	9,32,5,13
novel_circ_0012305	7.015725	0.000435	0.046225	Up	8	Slc20a2	ENSMUSG00000037656	23025034	23035616	+	10582	197	exon: 23035520–23035616	48,28,34

### 3.2 circRftn1 Was Upregulated Both In Vitro and In Vivo

**FIGURE 3:**
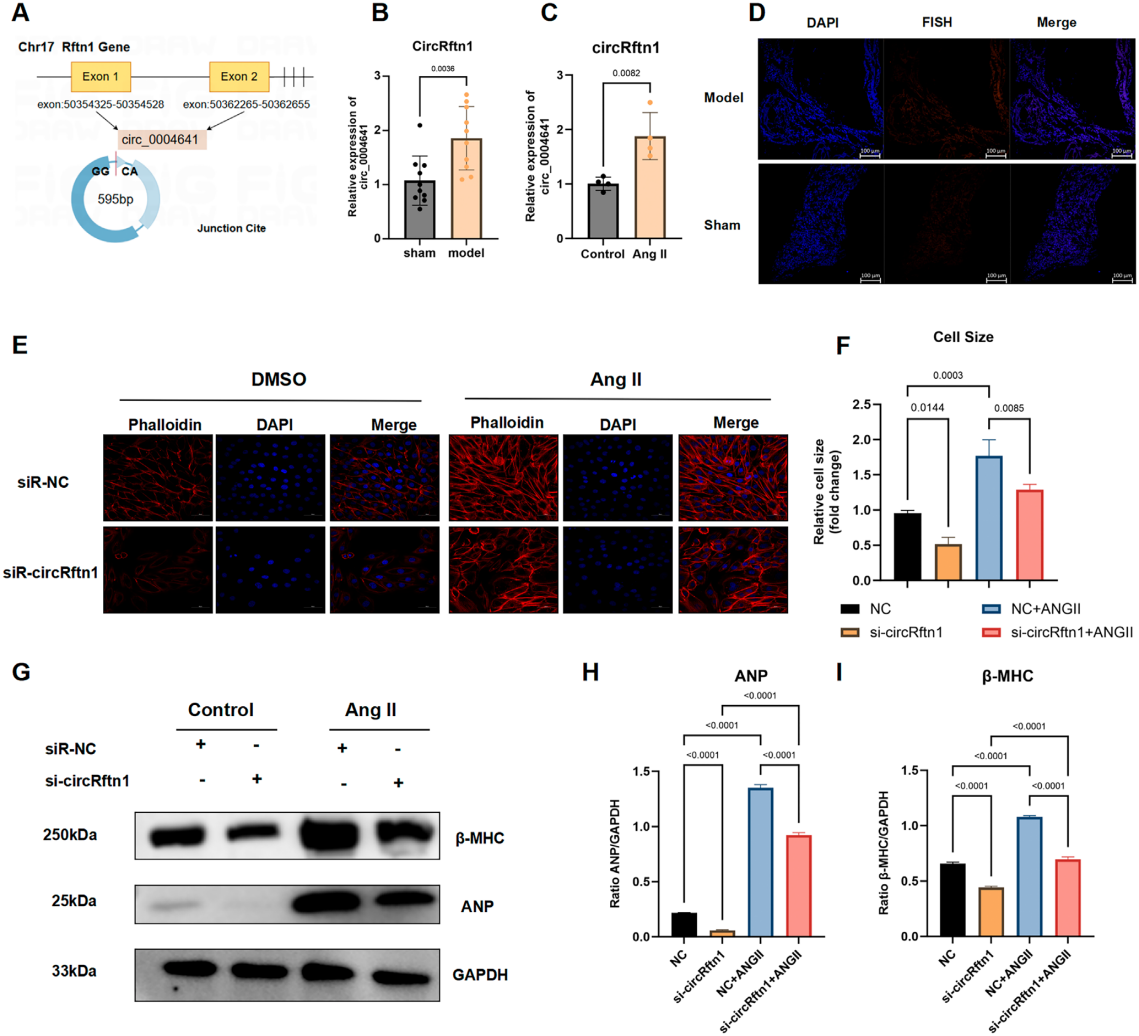
circRftn1 was upregulated in vivo and was correlated with cardiac hypertrophy. (A) circRftn1 was back‐spliced from the Rftn1 gene with 595 bp; (B, C) Compared to the control group, circRftn1 had a significant rise both in mouse ventricular myocardium (*N* = 10) and Ang II‐induced cardiomyocytes (*N* = 4); (D) RNA FISH for circRNA_004641 in mouse myocardium. circRftn1 was shown in orange, and nuclei were stained with DAPI; (E, F) Representative Phalloidin staining of NMVCs in both DMSO and Ang II co‐culture with or without siRNA transfection (*N* = 4); (G–I) Western blot assay of cardiac‐specific peptide and quantitative analysis showed the transfection of siRNA significantly reverses the expression of ANP and β‐MHC (*N* = 4). For presentation purposes, the GAPDH loading control shown in Figure 3G here is the same as in Figure 5C, as it represents the internal control for the same set of lysates run on parallel gels for different targets. Scale bar is 100 μm in (D) and 20 μm in (E). Data in (B), (C), (F), (H) and (I) were presented as mean ± SEM. For statistical analysis, 2‐tailed unpaired *t*‐test was used for (B) and (C); one‐way ANOVA with Bonferroni post hoc analysis was used for (F), (H) and (I). *p* < 0.05 was considered significant. Ang II, angiotensin II; ANOVA, analysis of variance; ANP, atrial natriuretic peptide; bp, base pairs; CircRftn1, circular RNA derived from the Rftn1 gene; DAPI, 4′,6‐diamidino‐2‐phenylindole; DMSO, dimethyl sulfoxide; NMVCs, neonatal mouse ventricular cardiomyocytes; RNA FISH, RNA fluorescence in situ hybridization; SEM, standard error of the mean; siRNA, small interfering RNA; β‐MHC, beta‐myosin heavy chain.

To validate the expression of selective candidate circRNAs, we performed RT‐qPCR validation on the remaining myocardial tissue used for sequencing. The results showed that only circRftn1 exhibited a significant increase in expression (Figure 3A,B), while the other molecules (circ_0009153, circ_0001213, circ_0009685, circ_0002423 and circ_0003046) showed no significant difference or a contradictory trend in expression (Figure S2D–H).

To support these results in animal tissues, hypertrophic cardiomyocytes were stimulated by Ang II. Phalloidin staining demonstrated hypertrophy of myofilaments and enlargement of cell size (Figure S3A,B), along with significant increases in the expression of β‐MHC and ANP (Figure S3C–E). Subsequently, qPCR validation showed that circRftn1 (Figure 3C) and circ_0009153 exhibited a matched increase in expression, while circ_0002423 presented a paradoxical trend compared to the sequencing results, and the other circRNAs showed no significant difference (Figure S3F). Therefore, we chose circRftn1 for further research due to its stable repeatability. FISH identified its subcellular location in the nucleus (Figure 3D).

To explore its correlation with cardiac hypertrophy, knockdown experiments were performed by transfecting with siRNA targeting circRftn1. The results showed that inhibiting circRftn1 significantly reversed the enlargement of cardiomyocytes in both the control and Ang II‐treated groups (Figure 3E,F), along with a decreased transcriptional level of ANP and β‐MHC (Figure S3G–I). Western blot and quantitative analysis also showed a sharp decrease in ANP and β‐MHC after transfection (Figure 3G–I). These results strongly indicate a relation between circRftn1 and cardiac hypertrophy.

### 3.3 miRNA‐1192 Was a Downstream Target of circRftn1 and Negatively Associated With Myocardial Hypertrophy

**FIGURE 4:**
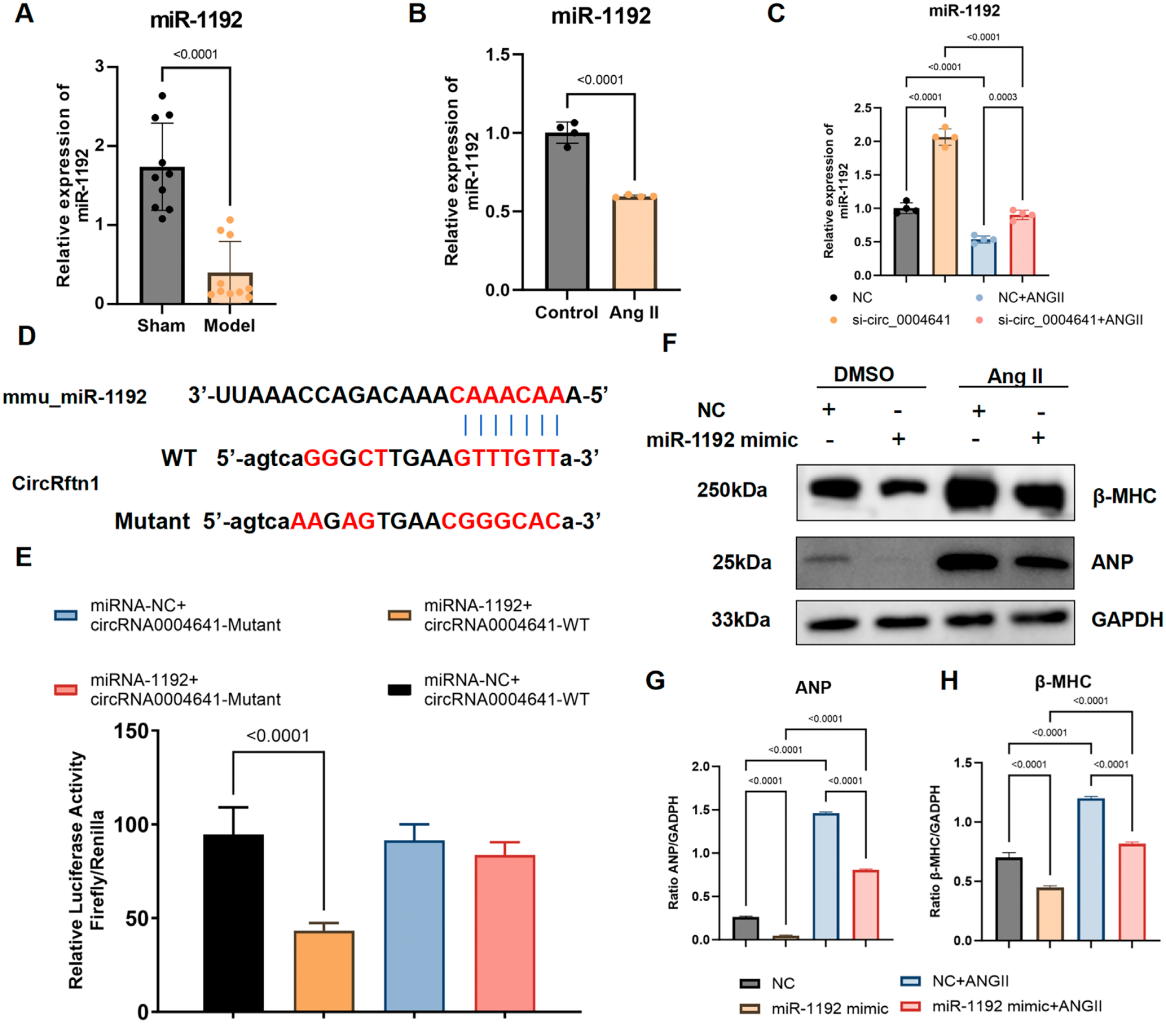
miR‐1192 was the downstream target of circRNAs and was correlated with cardiac hypertrophy. (A, B) miR‐1192 was downregulated both in hypertrophic myocardium (*N* = 10) and cardiomyocytes (*N* = 4); (C) By transfection with siRNA‐circRftn1, miR‐1192 had a significant upregulation in cardiomyocytes (both in the control and Ang II groups (*N* = 4)); (D, E) Dual luciferase reporter assay illustrated that only miRNA‐1192 and circRftn1 reaction has strong reduction on luciferase expression (*N* = 9); (F, G) Western blot assay and quantitative analysis showed that by co‐culture with miR‐1192 mimics, cardiac‐specific peptide of ANP and β‐MHC significantly decrease both in the control and Ang II groups (*N* = 4). For presentation purposes, the GAPDH loading control shown here is the same in Figures 4F and 5F, as it represents the internal control for the same set of lysates run on parallel gels for different target. Data in (A–C), (F), (G) and (H) were presented as mean ± SEM. For statistical analysis, 2‐tailed unpaired *t*‐test was used for (A) and (B); one‐way ANOVA with Bonferroni post hoc analysis was used for (C), (F), (G) and (H). *p* < 0.05 was considered significant. Ang II, angiotensin II; ANOVA, analysis of variance; ANP, atrial natriuretic peptide; circRftn1, circular RNA derived from the Rftn1 gene; MiR‐1192, microRNA‐1192; SEM, standard error of the mean; siRNA, small interfering RNA; β‐MHC, beta‐myosin heavy chain.

miR‐1192 was the only molecule regulated by circRftn1, as detected by miRanda (Figure 2E; Data Availability Statement). We observed a significant decrease in miR‐1192 both in the myocardium and NMVCs (Figure 4A,B). After knocking down circRftn1 via siRNA transfection, qPCR results demonstrated a significant increase in miR‐1192 expression, along with downregulation of ANP and brain natriuretic peptide (Figure 4C; Figure S3G,H) in NMVCs, indicating a potential regulatory relationship between circRftn1 and miR‐1192. To clarify the interaction between these two molecules, we performed dual luciferase reporter assays. In a 293T engineered cell system, we observed a strong interaction between the wild‐type‐circRftn1‐3'UTR and miR‐1192, while the mutant‐circRftn1‐3'UTR did not interact with miR‐1192 (Figure 4D,E). Therefore, we confirmed that circRftn1 and miR‐1192 interact through a sponge‐like mechanism, as we hypothesised.

To explore the functional role of miR‐1192 further, we co‐cultured NMVCs with miR‐1192 mimics. The results showed that miR‐1192 plays an inhibitory role in cardiac hypertrophy, as the transcriptional expression of ANP and β‐MHC dropped dramatically in both the control and Ang II treatment groups (Figure 4F–H). Whether knocking down circRftn1 or overexpressing miR‐1192 reversed cardiac hypertrophy in vitro, we hypothesise that the circRftn1/miR‐1192 axis comprises an underlying regulatory network in cardiac hypertrophy.

### 3.4 TRIM25 and TRIM41 Act as Downstream mRNA Targets in the ceRNA Network

**FIGURE 5:**
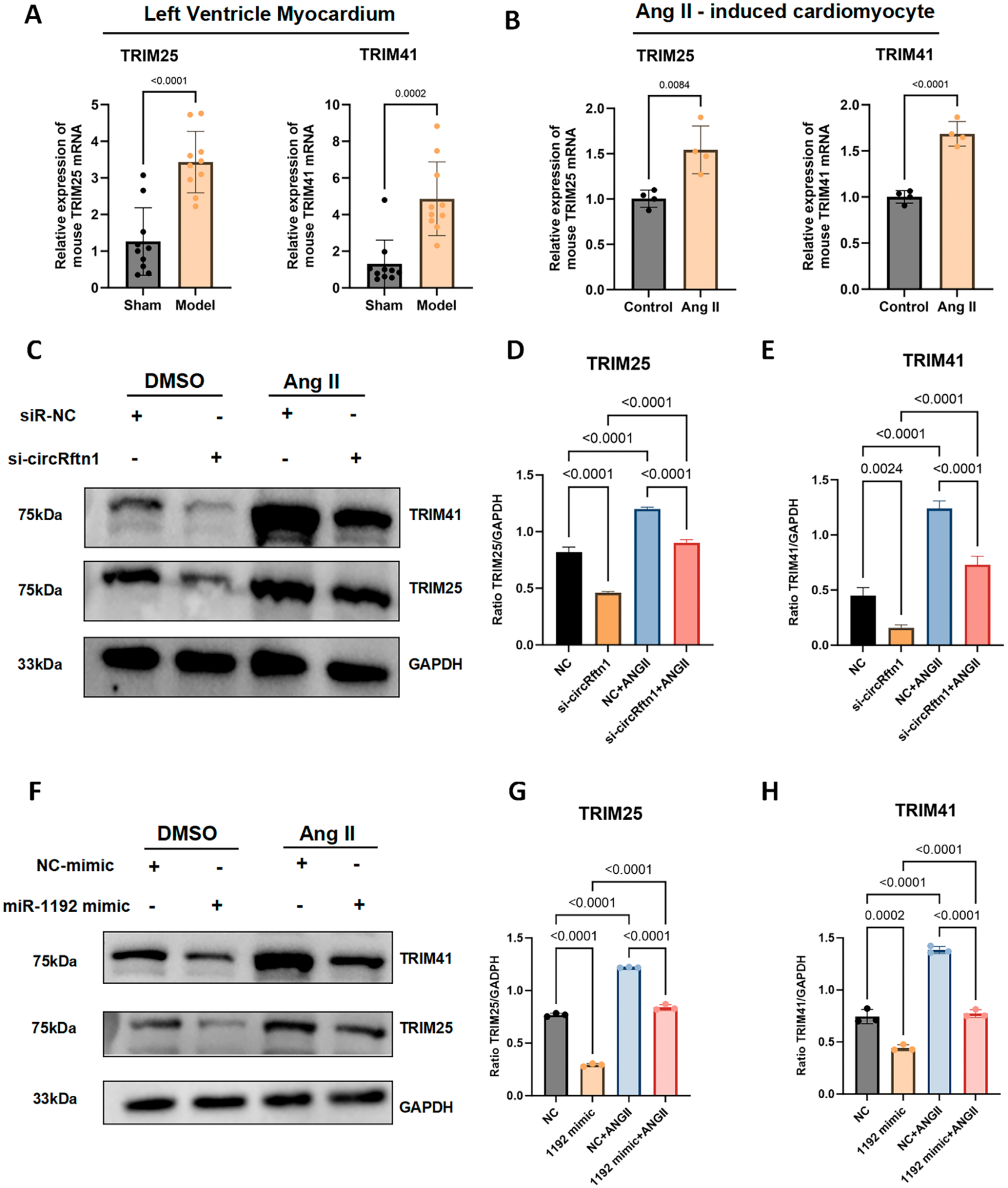
TRIM25 and TRIM41 are downstream mRNAs regulated by the circRftn1/miR‐1192 axis. (A, B) TRIM25 and TRIM41 were up‐regulated both in myocardium (*N* = 10) and NMVCs (*N* = 4); (C–E) Western blot showed that, after transfected with siRNA, TRIM25, and TRIM41 had a significant decrease in cardiomyocytes (both in the control and Ang II groups) (*N* = 4); (F–H) Western blot and quantitative analysis illustrated that, after transfection with miR‐1192 mimics, TRIM25 and TRIM41 showed a significant decrease in cardiomyocytes (both in the control and Ang II groups) (*N* = 4). The GAPDH Gel in Figure 5C,F is parallel to the Gel in Figures 3G and 4F, separately, as illustrated in the Figures 3 and 4 legend. Data in (A), (B), (D), (E), (G) and (H) were presented as mean ± SEM. For statistical analysis, 2‐tailed unpaired *t*‐test was used for (A) and (B); one‐way ANOVA with Bonferroni post hoc analysis was used for (D), (E), (G) and (H). *p* < 0.05 was considered significant. Ang II, angiotensin II; ANOVA, analysis of variance; miR‐1192, microRNA‐1192; NMVCs, neonatal mouse ventricular cardiomyocytes; SEM, standard error of the mean; siRNA, small interfering RNA; TRIM25, tripartite motif‐containing protein 25; TRIM41, tripartite motif‐containing protein 41.

Through the **miRDB** website, we identified 14 potential downstream mRNAs regulated by miRNA‐1192, including members of the TRIM protein family (TRIM6, TRIM21, TRIM25, TRIM32, TRIM33, TRIM41, TRIM71), the ring finger (RNF) protein family (RNF13, RNF138, RNF139, RNF169, RNF170), Dtx31 and RAD18. To verify these targets, RT‐qPCR was conducted at both the animal and cell levels, and the results showed that TRIM25 and TRIM41 exhibited a stable increase in expression (Figure 5A,B). Other mRNAs were also detected but displayed non‐stable expression trends (Figures S4A–L and S5A–E), indicating that TRIM25 and TRIM41 are reliable molecules regulated by the circRftn1/miRNA‐1192 axis in cardiac hypertrophy. We then transfected siRNA and co‐cultured cardiomyocytes with miR‐1192 mimics. The results demonstrated that TRIM25 and TRIM41 were significantly downregulated at both the transcriptional and translational levels after si‐circRftn1 transfection (Figure 5C–E; Figure S5F,G), which was contrary to the previous expression trends. Next, we co‐cultured NMVCs with miR‐1192 mimics, and as expected, the expression of TRIM25 and TRIM41 decreased sharply in both the control and Ang II‐treated groups (Figure 5F–H). Based on these findings, we conclude that overexpression of circRftn1 can competitively absorb miR‐1192, thereby suppressing the inhibitory role of miR‐1192 on the expression of TRIM25 and TRIM41 in cardiac hypertrophy.

### 3.5 NF‐κB May Act as an Underlying Key Pathway in the Regulation of Cardiac Hypertrophy

**FIGURE 6:**
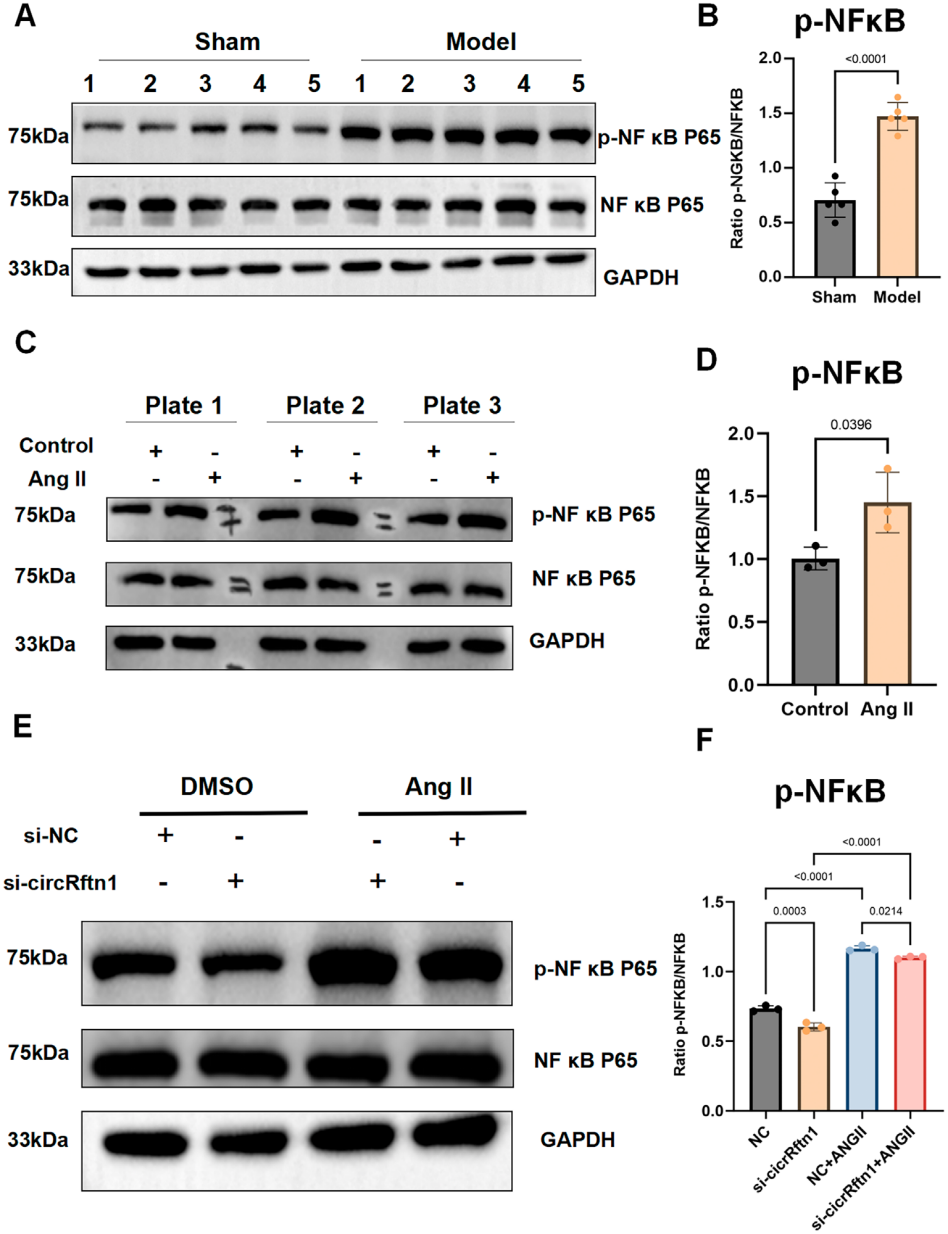
NF‐κB signalling pathway may play a regulatory role through the ceRNA network on cardiac hypertrophy. (A, B) Compared to the sham group, NF‐κB signalling pathway had a significant increase in model mice after AAC surgery (*N* = 5); (C, D) Compared to the control group, NF‐κB signalling pathway had a significant increase in Ang II‐induced cardiomyocytes (*N* = 3); (E, F) After transfection of si‐circRftn1, P65 and p‐P65 had been significantly suppressed both in DMSO‐ and Ang II‐induced cardiomyocytes (*N* = 3). Data in (B), (D) and (F) were presented as mean ± SEM. For statistical analysis, 2‐tailed unpaired *t*‐test was used for (B); one‐way ANOVA with Bonferroni post hoc analysis was used for (D) and (F). *p* < 0.05 was considered significant. AAC, abdominal aortic constriction; Ang II, angiotensin II; ANOVA, analysis of variance; ceRNA, competing endogenous RNA; DMSO, dimethyl sulfoxide; NF‐κB, nuclear factor kappa‐light‐chain‐enhancer of activated B cells; P65, subunit of NF‐κB; p‐P65, phosphorylated P65; SEM, standard error of the mean; si‐circRftn1, small interfering RNA targeting circRftn1.

Previous studies have reported the regulation of the NF‐κB signalling pathway by non‐coding RNA in myocardial hypertrophy [45, 46]. Western blot and quantitative analysis indicated that, compared to the control group, there was an increased expression of p65 and phosphorylated p65 (p‐p65) protein in mouse myocardial ventricular tissue (Figure 6A,B). Repeated results were observed in a cell model, with the same expression trend (Figure 6C,D). By knocking down the expression of circRftn1, we found that, while the expression in the Ang II group was higher than in the control group, both groups showed a significant decrease in the expression of p65 and p‐p65 protein after siRNA transfection (Figure 6E,F). This suggests that the circRftn1/miR‐1192 axis may play an important role in the activation of the NF‐κB signalling pathway in cardiac hypertrophy, though the underlying mechanism requires further research. In addition, we also investigated the TGFβ1/SMAD signalling pathway and FN1 protein (Figure S6A–F), which displayed the same expression trend as NF‐κB.

In conclusion, we identified a novel circRftn1 via sequencing and constructed a regulatory network through the circRftn1/miR‐1192‐TRIM25/TRIM41 axis in cardiac hypertrophy. In addition, NF‐κB may be the downstream signalling pathway involved in this regulatory mechanism.

## 4 Discussion

CircRNAs, which are non‐coding sequences without protein‐encoding function, have recently been recognised for their considerable regulatory roles since their discovery [27, 47]. As key molecules in the cardiovascular field, the identification of these molecules facilitates a comprehensive understanding of pathological myocardial hypertrophy, with a growing number of ceRNA networks being uncovered [32, 38, 48, 49].

In our study, a novel mmu_circ_0004641 (which we named circRftn1 based on its parental gene, following canonical naming rules [29, 50]) and its associated ceRNA network (circRftn1/miR‐1192) were characterised in the context of cardiac hypertrophy. Firstly, we verified the interaction between circRftn1 and miR‐1192 and established the sponge‐like effect of circRftn1 in the regulation of cardiac hypertrophy. We then identified the downstream mRNA regulated by the circRftn1/miRNA‐1192 axis. Bioinformatic analysis predicted ubiquitination as a key mechanism, leading us to focus on TRIM25/TRIM41. Our study showed that the circRftn1/miRNA‐1192 axis promotes the expression of TRIM25 and TRIM41 in pathological cardiac hypertrophy. The TRIM family [51, 52], known as E3 ligases, plays prominent roles in cardiac hypertrophy by activating downstream signalling pathways involved in the pathology of the disease (such as TRIM8 [53], TRIM32 [54], TRIM44 [55], TRIM63 [56], and others). Previous studies have revealed the unique function of TRIM25 [57] and TRIM41 [58, 59], as activators targeting the downstream NF‐κB pathway at the K63 tyrosine site, thereby enhancing or inhibiting tumour proliferation and metastasis. The regulation of the NF‐κB pathway by non‐coding RNA has been previously reported [46]. We verified the expression of p65, regulated by circRftn1, which was speculated to be part of this process. However, the precise mechanism of how TRIM25 and TRIM41 regulate cardiac hypertrophy and their underlying correlation with p65 protein remains unknown and requires further investigation. Moreover, other signalling pathways, such as TGFβ or FN1, also warrant exploration.

There are some limitations in our study worth noting: (1) The miRNAs and mRNAs we verified were predicted using the miRanda and miRDB databases. To ensure the accuracy of the study, we performed repeated validation experiments at both in vivo and in vitro levels; (2) The relationship between TRIM proteins and the NF‐κB signalling pathway still requires validation through techniques such as co‐immunoprecipitation or immunoprecipitation‐mass spectrometry. Further studies are needed to comprehensively verify the amino‐acid binding sites for ubiquitination and the underlying regulatory mechanisms; (3) Although we identified 30 differentially expressed circRNAs, we may not have captured all of them due to limited myocardium tissue samples. We selectively chose the top two upregulated and top four downregulated circRNAs for further study, which could overlook the potential roles of other molecules with differential expression; (4) Lastly, to more accurately assess the role of circRftn1, standard assays such as back‐splice junction PCR or Sanger sequencing should be included in further animal rescue studies.

## 5 Conclusion

We reported an incremental ceRNA network (circRftn1/miRNA‐1192) in myocardial hypertrophy. Mechanistically, upregulated circRftn1 sponged miR‐1192 by inhibiting its regulatory role on TRIM25 and TRIM41, which contribute to the progression of myocardial hypertrophy. The underlying regulation of myocardial hypertrophy by TRIM25/TRIM41 proteins via the NF‐κB pathway requires in‐depth functional verification (see Graphical Abstract, created by FigDraw 2.0, ID: ATWYR59d98).

## Author Contributions

**Guangcheng Liu:** conceptualization (equal), formal analysis (equal), methodology (equal), validation (lead), writing – original draft (lead). **Haipeng Zhang:** data curation (equal), formal analysis (equal), methodology (supporting), validation (supporting). **Jingdai Zhang:** data curation (equal), formal analysis (equal), methodology (equal). **Hao Qian:** data curation (equal), formal analysis (equal). **Liang Wang:** data curation (equal), formal analysis (equal). **Lianfeng Chen:** data curation (supporting), validation (supporting), writing – review and editing (supporting). **Zhujun Shen:** conceptualization (equal), funding acquisition (lead), writing – review and editing (lead).

## Ethics Statement

The study was conducted according to the guidelines of the Declaration of Helsinki and was approved by the Ethics Committee of Peking Union Medical College Hospital on April 15, 2024 (PUMCH, XHDW‐24‐86, China).

## Conflicts of Interest

The authors declare no conflicts of interest.

## Supporting information


**Figure S1:** Construction of the AAC animal model.
**Figure S2:** Identification of CircRNA molecules in the myocardium.
**Figure S3:** Identification of circRftn1 in NMVCs.
**Figure S4:** qPCR results of target mRNAs in mouse ventricle tissue.
**Figure S5:** qPCR validation results of target mRNA in vitro.
**Figure S6:** Other underlying signalling pathways involved in the ceRNA regulatory network.

## Data Availability

The original sequencing datasets generated during the current study, including cirRNA sequences and the circRNA‐miRNA pair set, are available in the Mendeley Database repository (Mendeley Data, V1, https://doi.org/10.17632/hy5pmx39g5.1).
