# Supplementary material for: Circular RNA Rftn1 Promotes Cardiac Hypertrophy In Vitro and In Vivo by Sponging miRNA‐1192 to Upregulate Tripartite Motif Protein 25 and 41
Source: J Cell Mol Med. 2025 Oct 13;29(19):e70892. doi: 10.1111/jcmm.70892 (PMC12516353; doi:10.1111/jcmm.70892)
Supplement: Supplementary file 1 — Figure S1: Construction of the AAC animal model. Figure S2: Identification of CircRNA molecules in the myocardium. Figure S3: Identification of circRftn1 in NMVCs. Figure S4: qPCR results of target mRNAs in mouse ventricle tissue. Figure S5: qPCR validation results of target mRNA in vitro. Figure S6: Other underlying signalling pathways involved in the ceRNA regulatory network. [file JCMM-29-e70892-s001.zip › JCMM_70892_supinfo.docx]

**Figure S1. Construction of the AAC animal model**

**(A, B)** Macroscopic observation of mice heart and heart mass shows a significant increase in the surgical group (N = 8); **(C, D)** Compared to the sham group, the ventricle diameter and ventricle cavity diameter show a significant difference (N = 3); **(E)** Representative M-mode echocardiography image for each mouse; **(F)–(K)** Other echocardiography evaluation on cardiac parameters: Interventricular septal thickness, end-diastolic /end-systolic (IVS;d /IVS;s); left ventricular internal diameter, end-diastolic/end-systolic (LVID;d / LVID;s); left ventricular volume, end-diastolic/end-systolic (LV Vol;d / LV Vol;s). Compared to the sham group, the surgical group shows a significant decrease (N = 8). Data in (**C, D, and F–K)** are presented as mean ± SEM. For statistical analysis, 2-tailed unpaired t-test was used for **C, D, and F–K**. *P*＜0.05 was considered significant.

**Abbreviations**: AAC: abdominal aortic constriction; IVS;d / IVS;s: interventricular septal thickness in end-diastole/end-systole; LVID;d / LVID;s: left ventricular internal diameter in end-diastole/end-systole; LV Vol;d / LV Vol;s: left ventricular volume in end-diastole/end-systole; SEM: standard error of the mean.

**Figure S2. Identification of CircRNA molecules in the myocardium**

**(A)** TPM box plot for gene expression in each mouse; **(B)** Box plot for circRNAs distribution in each mouse; **(C)** Chromosomal distribution of differentially expressed circRNAs in each mouse of sham and model groups; **(D)** Validation in mouse left myocardium: compared to the control group, circ_009153, circ_0001213, and circ_009685 molecules showed no difference in mouse ventricular muscle tissue. Although circ_003046 and circ_0002423 show a significant increase, this change is contrary to sequencing results (N = 10). Data in (**D–K)** are presented as mean ± SEM. For statistical analysis, 2-tailed unpaired t-test was used for **D–K**. *P*＜0.05 was considered significant.

**Abbreviations**: circRNA: circular RNA; TPM: transcripts per million; SEM: standard error of the mean

**Figure S3. Identification of circRftn1 in NMVCs**

**(A, B)** Ang-II induced hypertrophic cardiomyocytes showed a significant increase in cell size compared to the control group (N = 3); **(C)–(E)** Western blot illustrated a significant increase of β-MHC and ANP in Ang II induced NMCVs (N = 3). **(F)** Validation in Ang II-induced hypertrophic cardiomyocytes: Compared to the control group, circ_009153, circ_0003046, circ_0001213, and circ_009685 molecules showed no difference in Ang II cocultured NMVCs. Although circ_002423 shows a significant increase, this change is contrary to sequencing results (N = 4); **(G, H)** qPCR validation results showed that after transfection of si-circRftn1, ANP and β-MHC showed a significant decrease both in the control and Ang II-induced groups (N = 4). Scale bar is 100 um. Data in (**B, D, E, G, and H)** are presented as mean ± SEM. For statistical analysis, 2-tailed unpaired t-test was used for **B, D, and E**. *P*＜0.05 was considered significant.

**Abbreviations**: circRNA: circular RNA; Ang II: angiotensin II; β-MHC: beta-myosin heavy chain; ANP: atrial natriuretic peptide; NMVCs: neonatal mouse ventricular cardiomyocytes; qPCR: quantitative polymerase chain reaction; si-circRftn1: small interfering RNA targeting circRftn1; SEM: standard error of the mean.

**Figure S4. qPCR results of target mRNAs in mouse ventricle tissue**

**(A)-(F)** Compared to the control group, RNF13, TRIM6, TRIM21, TRIM32, TRIM71, and Dtx3l molecules showed significant difference in mouse ventricular muscle tissue (N = 4); **(G) - (L)** Compared to the control group, RNF138, RNF139, RNF169, RNF170, TRIM33, and Rad18 molecules showed no difference in mouse ventricular muscle tissue (N = 4). Data in (**A-L)** are presented as mean ± SEM. For statistical analysis, 2-tailed unpaired t-test was used for **A-L**. *P*＜0.05 was considered significant.

**Abbreviations**: qPCR: quantitative polymerase chain reaction; RNF13: ring finger protein 13; TRIM6: tripartite motif-containing 6; TRIM21: tripartite motif-containing 21; TRIM32: tripartite motif-containing 32; TRIM71: tripartite motif-containing 71; Dtx3l: Deltex E3 ubiquitin ligase 3-like; RNF138: ring finger protein 138; RNF139: ring finger protein 139; RNF169: ring finger protein 169; RNF170: ring finger protein 170; TRIM33: tripartite motif-containing 33; Rad18: RAD18 homolog; SEM: standard error of the mean.

**Figure S5. qPCR validation results of target mRNA *in vitro***

**(A)-(E)** Compared to the control group, TRIM21, TRIM25, TRIM41, TRIM32, TRIM71, and Dtx3l molecules showed significant difference in Ang-II induced cardiomyocytes (N = 4); (F, G) By transfection of si-circRftn1, transcriptional expression of TRIM25 and TRIIM41 showed a significant decrease both in the control and Ang II-induced groups (N = 4). Data in (**A-E, F, and G)** are presented as mean ± SEM. For statistical analysis, 2-tailed unpaired t-test was used for **A-E;** one-way ANOVA with Bonferroni post hoc analysis was used for **F and G**. *P*＜0.05 was considered significant.

**Abbreviations**: qPCR: quantitative polymerase chain reaction; TRIM21: tripartite motif-containing 21; TRIM25: tripartite motif-containing 25; TRIM41: tripartite motif-containing 41; TRIM32: tripartite motif-containing 32; TRIM71: tripartite motif-containing 71; Dtx3l: Deltex E3 ubiquitin ligase 3-like; si-circRftn1: small interfering RNA targeting circRftn1; SEM: standard error of the mean; ANOVA: analysis of variance.

**Figure S6. Other underlying signaling pathways involved in the ceRNA regulatory network**

1. -(F) Other signaling pathways (TGFβ-SMAD pathway) also showed relatively low expression after siRNA transfection, both in DMSO- and Ang II-induced NMVCs (N = 4). Data in (**B, C, E, and F)** are presented as mean ± SEM. For statistical analysis, one-way ANOVA with Bonferroni post hoc analysis was used for **B, C, E, and F.** *P*＜0.05 was considered significant.

**Abbreviations**: siRNA: small interfering RNA; DMSO: dimethyl sulfoxide; Ang II: angiotensin II; NMVCs: neonatal mouse ventricular cardiomyocytes; TGFβ: transforming growth factor beta; SMAD: a family of proteins involved in signal transduction; ANOVA: analysis of variance; SEM: standard error of the mean.
