# Supplementary figures and images for: Circular RNA Rftn1 Promotes Cardiac Hypertrophy In Vitro and In Vivo by Sponging miRNA‐1192 to Upregulate Tripartite Motif Protein 25 and 41
Source: J Cell Mol Med. 2025 Oct 13;29(19):e70892. doi: 10.1111/jcmm.70892 (PMC12516353; doi:10.1111/jcmm.70892)

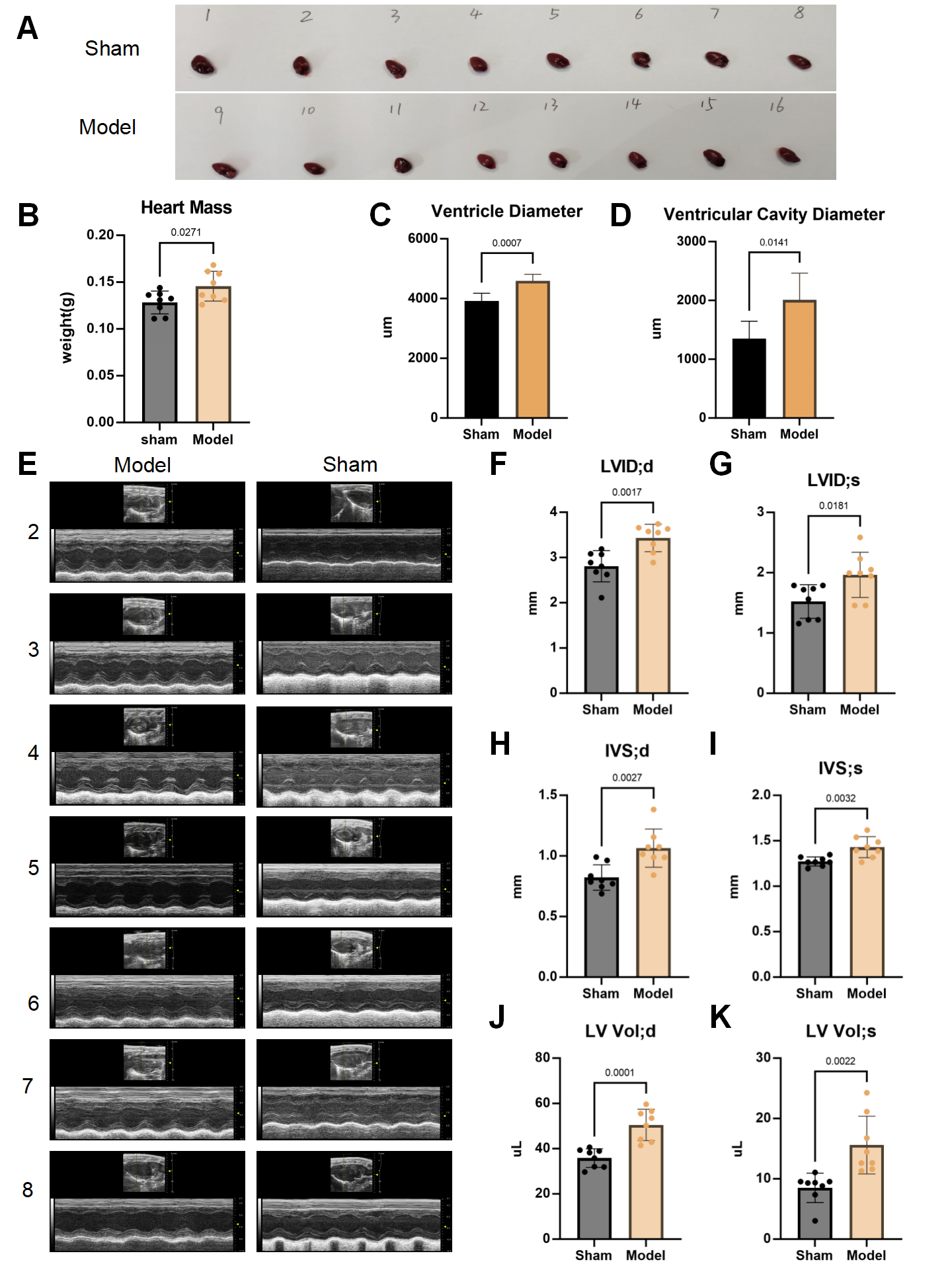

Supplement: Supplementary file 1 — Figure S1: Construction of the AAC animal model. Figure S2: Identification of CircRNA molecules in the myocardium. Figure S3: Identification of circRftn1 in NMVCs. Figure S4: qPCR results of target mRNAs in mouse ventricle tissue. Figure S5: qPCR validation results of target mRNA in vitro. Figure S6: Other underlying signalling pathways involved in the ceRNA regulatory network. [file JCMM-29-e70892-s001.zip › jcmm70892-sup-0001-FigureS1@Figure S1.tif]

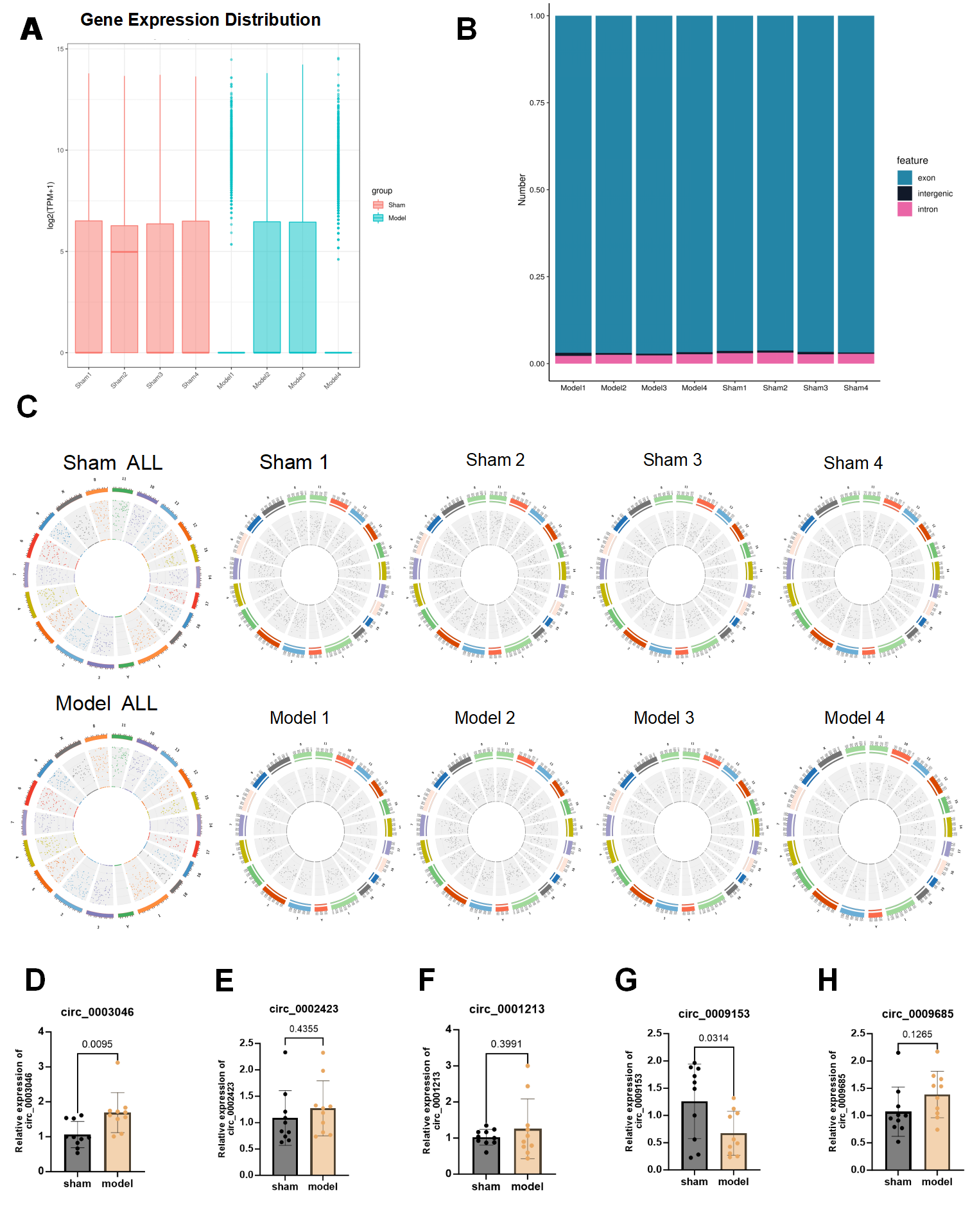

Supplement: Supplementary file 1 — Figure S1: Construction of the AAC animal model. Figure S2: Identification of CircRNA molecules in the myocardium. Figure S3: Identification of circRftn1 in NMVCs. Figure S4: qPCR results of target mRNAs in mouse ventricle tissue. Figure S5: qPCR validation results of target mRNA in vitro. Figure S6: Other underlying signalling pathways involved in the ceRNA regulatory network. [file JCMM-29-e70892-s001.zip › jcmm70892-sup-0002-FigureS2@Figure S2.tif]

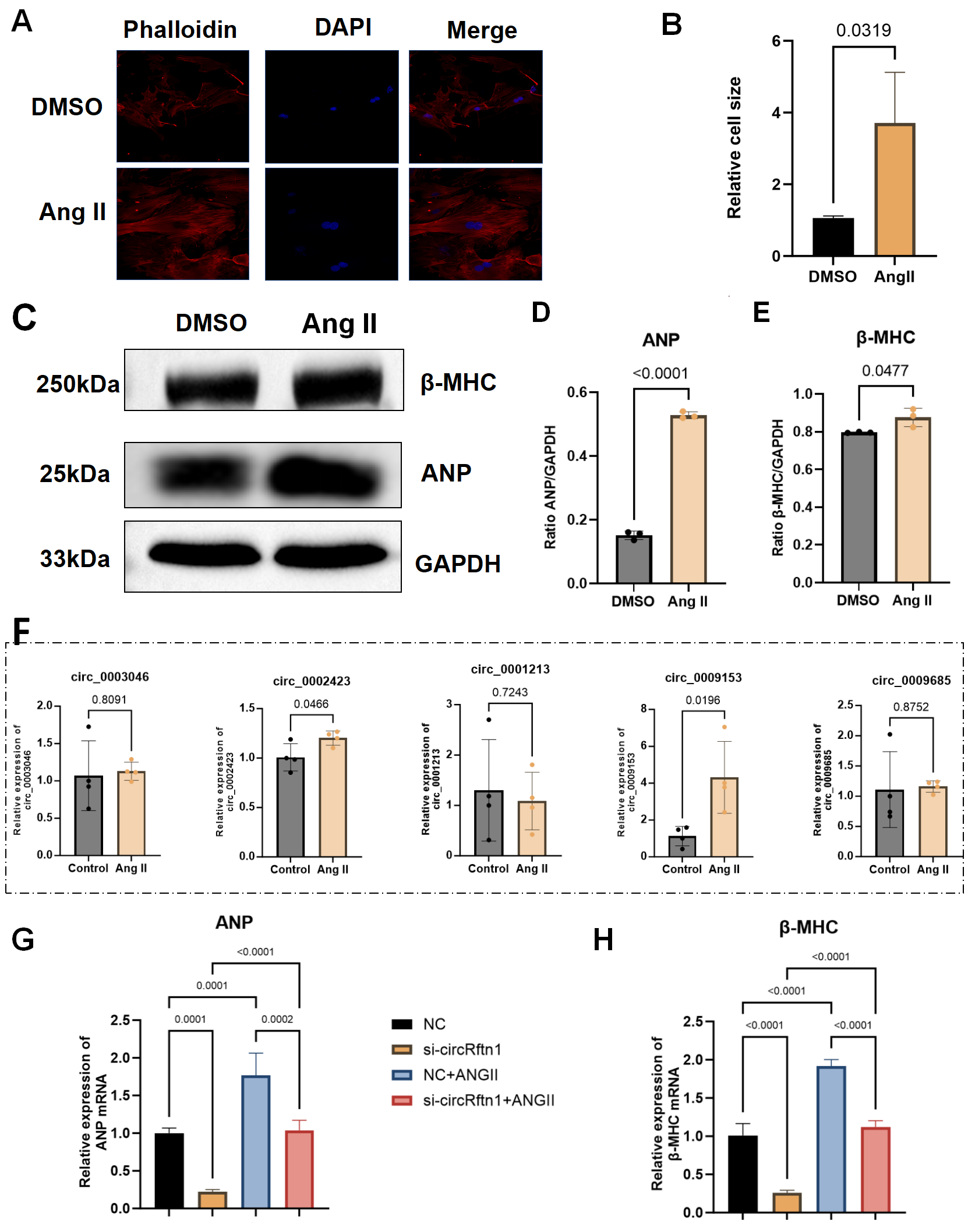

Supplement: Supplementary file 1 — Figure S1: Construction of the AAC animal model. Figure S2: Identification of CircRNA molecules in the myocardium. Figure S3: Identification of circRftn1 in NMVCs. Figure S4: qPCR results of target mRNAs in mouse ventricle tissue. Figure S5: qPCR validation results of target mRNA in vitro. Figure S6: Other underlying signalling pathways involved in the ceRNA regulatory network. [file JCMM-29-e70892-s001.zip › jcmm70892-sup-0003-FigureS3@Figure S3.tif]

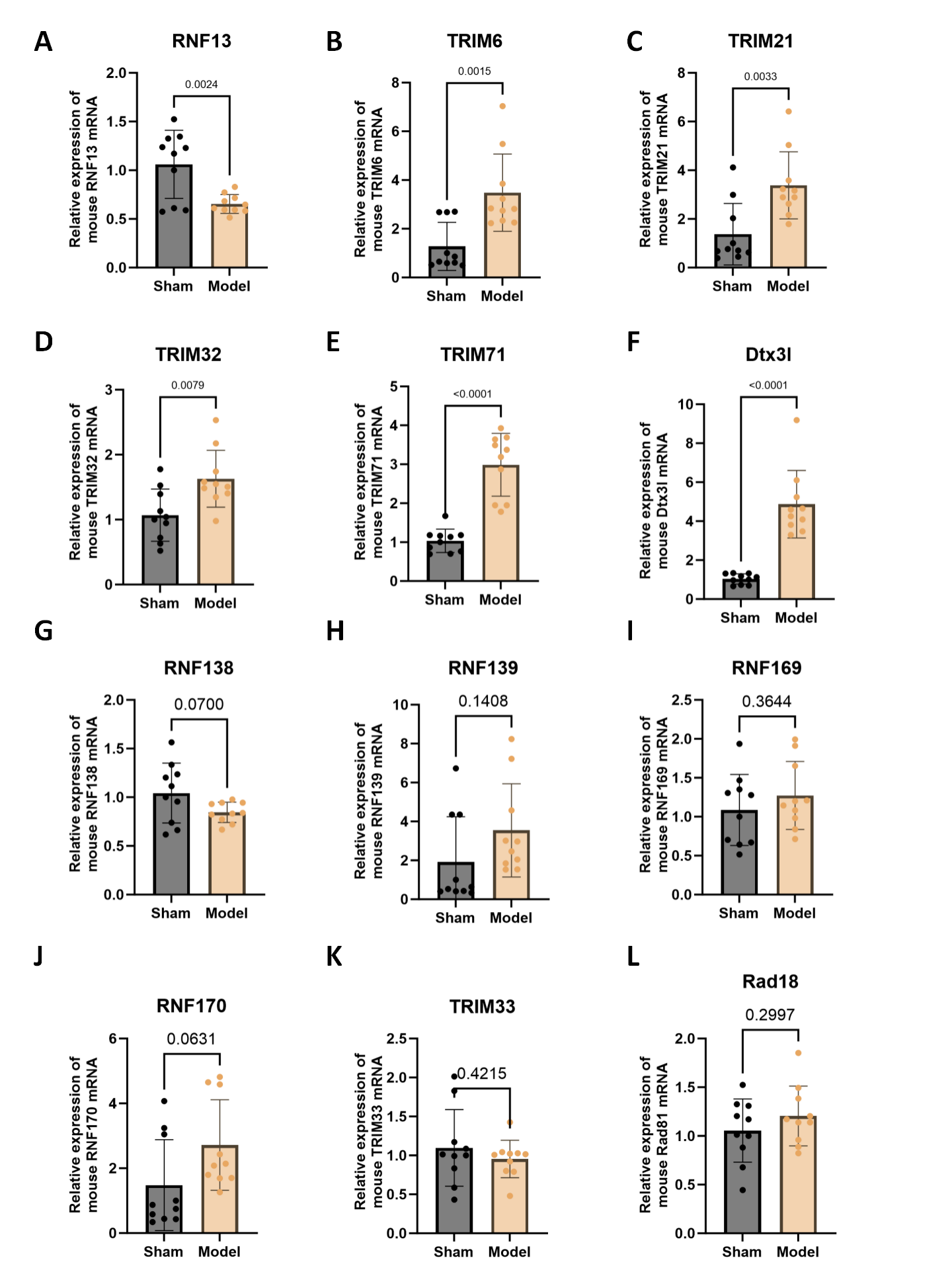

Supplement: Supplementary file 1 — Figure S1: Construction of the AAC animal model. Figure S2: Identification of CircRNA molecules in the myocardium. Figure S3: Identification of circRftn1 in NMVCs. Figure S4: qPCR results of target mRNAs in mouse ventricle tissue. Figure S5: qPCR validation results of target mRNA in vitro. Figure S6: Other underlying signalling pathways involved in the ceRNA regulatory network. [file JCMM-29-e70892-s001.zip › jcmm70892-sup-0004-FigureS4@Figure S4.tif]

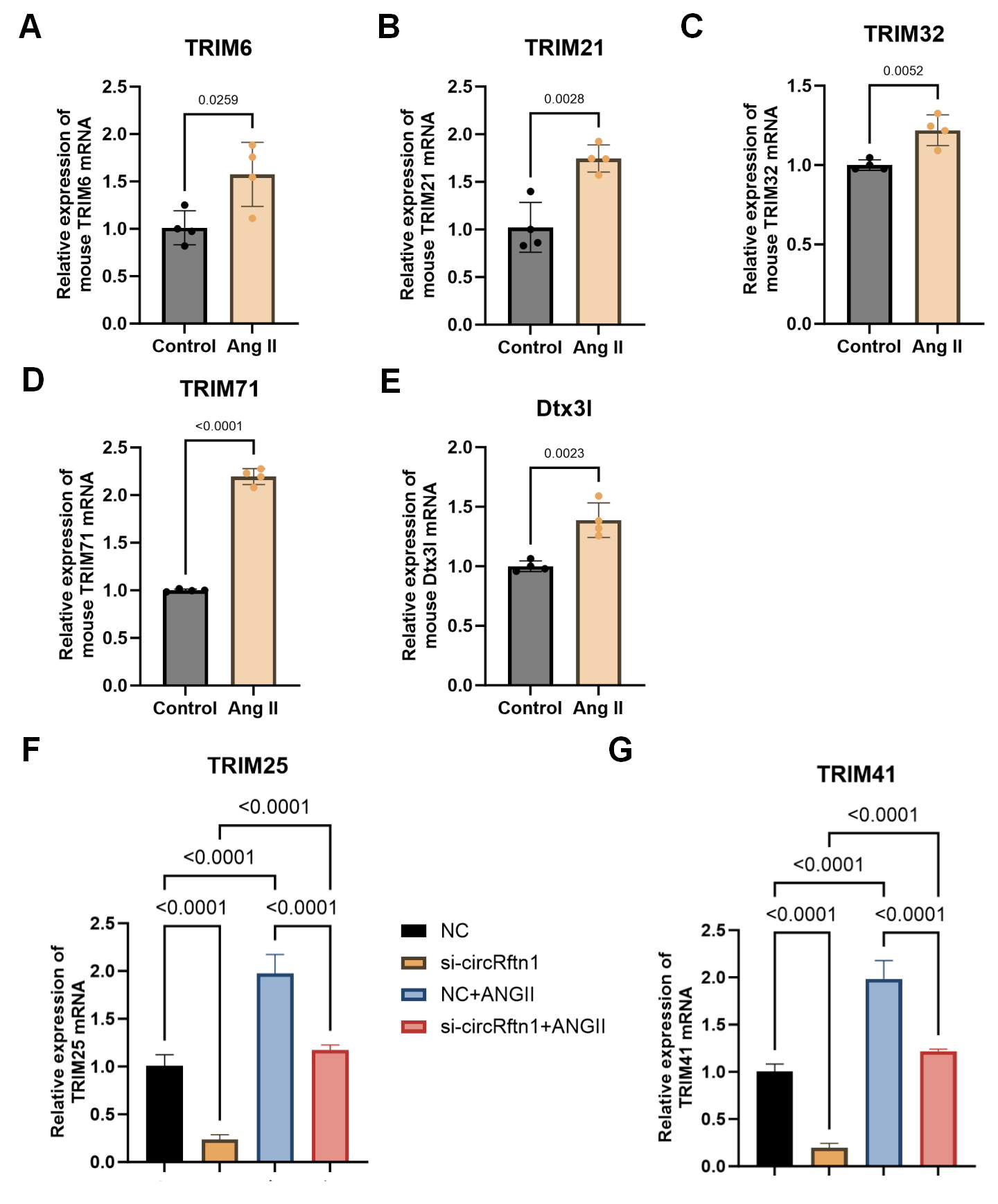

Supplement: Supplementary file 1 — Figure S1: Construction of the AAC animal model. Figure S2: Identification of CircRNA molecules in the myocardium. Figure S3: Identification of circRftn1 in NMVCs. Figure S4: qPCR results of target mRNAs in mouse ventricle tissue. Figure S5: qPCR validation results of target mRNA in vitro. Figure S6: Other underlying signalling pathways involved in the ceRNA regulatory network. [file JCMM-29-e70892-s001.zip › jcmm70892-sup-0005-FigureS5@Figure S5.tif]

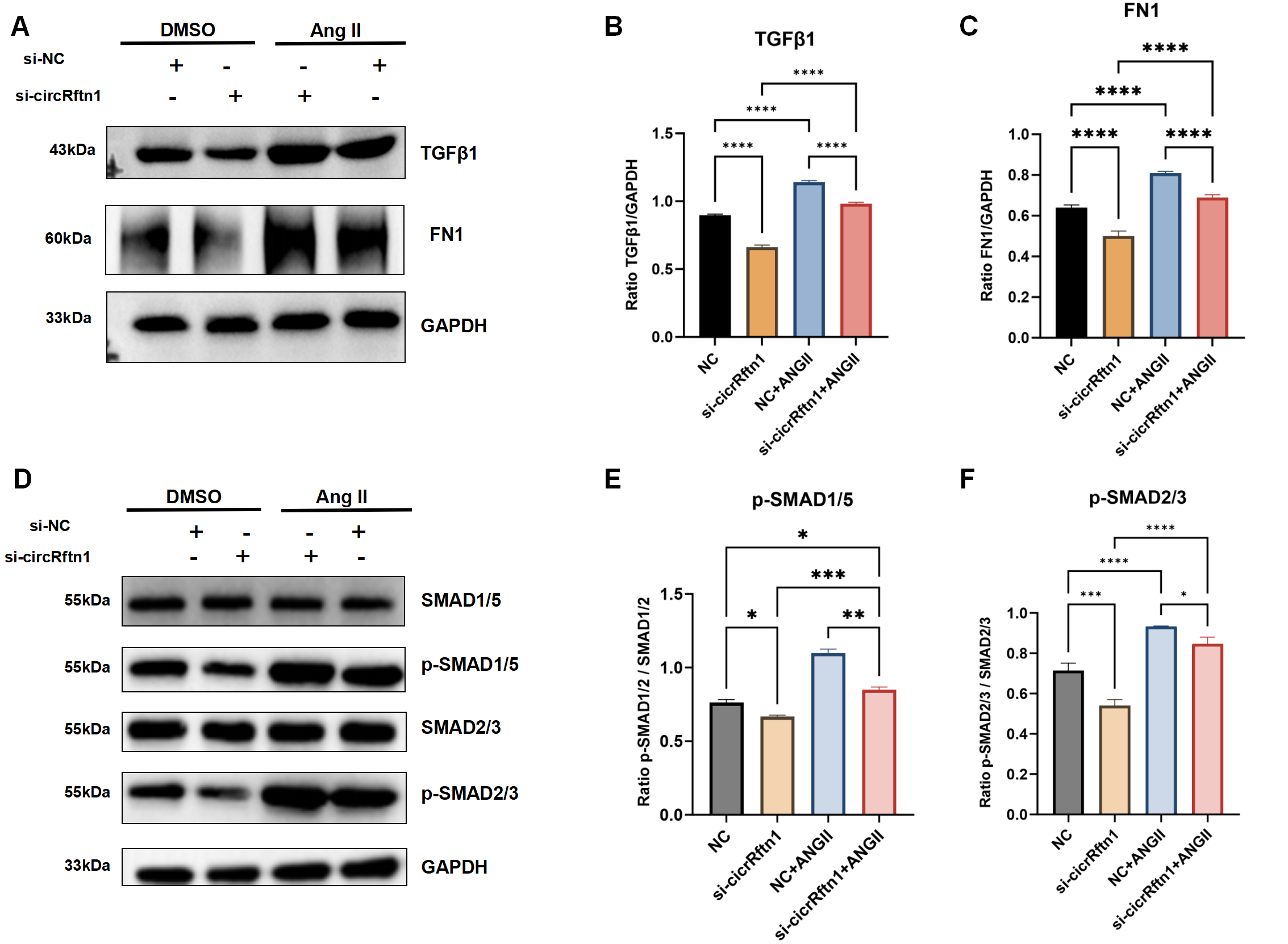

Supplement: Supplementary file 1 — Figure S1: Construction of the AAC animal model. Figure S2: Identification of CircRNA molecules in the myocardium. Figure S3: Identification of circRftn1 in NMVCs. Figure S4: qPCR results of target mRNAs in mouse ventricle tissue. Figure S5: qPCR validation results of target mRNA in vitro. Figure S6: Other underlying signalling pathways involved in the ceRNA regulatory network. [file JCMM-29-e70892-s001.zip › jcmm70892-sup-0006-FigureS6@Figure S6.tif]
